# Rare occurence of amennorrhea associated with olanzapine : a case report

**DOI:** 10.1192/j.eurpsy.2022.1848

**Published:** 2022-09-01

**Authors:** A. Khivsara, D. Goya, N. Nebhinani

**Affiliations:** All India Institute of Medical Sciences, Jodhpur, Psychiatry, Jodhpur, India

**Keywords:** Olanzapine, Hyperprolactinemia, Amenorrhea, Aripiprazole

## Abstract

**Introduction:**

Amenorrhea secondary to hyperprolactinemia is one of the frequent adverse effects associated with the use of atypical antipyschotics. It is often neglected but can interrupt the compliance of treatment. Several studies indicate that olanzapine does not significantly affect serum prolactin levels in the long term, although contrary has been observed in few case reports.

**Objectives:**

To report a case of olanzapine-induced amenorrhea due to hyperprolactinemia.

**Methods:**

A 27-year-old woman with history of stillbirth 5 months prior, presented to OPD with hallucinatory behaviour and socio-occupational dysfunction for 5 months. She was on tianeptine 12.5 mg, escitalopram 10 mg and alprazolam 0.5 mg at presentation and was having regular menses. On assessment, she was diagnosed with unspecified psychosis. Her ongoing medications were stopped and she was started on Olanzapine (optimized to 20 mg/day) after which she reported significant improvement however developed amenorrhea within next 2 months hence advised to consult Obgyn. Urine pregnancy test came out negative and prolactin level was found to be 64.2 ng/ml. Other investigations including MRI were within normal limit. Olanzapine was cross tapered with Aripiprazole (maintained at 10 mg/day). Clonazepam was advised SOS for anxiety.

**Results:**

After 1 month of aripiprazole treatment, monthly menses resumed and prolactin level returned to normal range. No biological dysfunction or other side effects were reported by the patient.

**Conclusions:**

Olanzapine-induced amennorhea secondary to hyperprolactinemia, is a rare but possible event. We report a case in which olanzapine induced amenorrhea normalized after switching to aripiprazole. Baseline prolactin level should be obtained as they help in the management of patients with neuroleptic-induced hyperprolactinemia.

**Disclosure:**

No significant relationships.

